# Gangrena Oris in a Python: A New Form of Bothrocephalus

**Published:** 1882-07

**Authors:** 


### CASE DEPARTMENT.

---

### 1.—GANGRENA ORIS IN A PYTHON—A NEW FORM OF BOTHROCEPHALUS.

In May last a large python died in the Central Park menagerie, after suffering some time from the ulcerated sore mouth common to serpents. At the post-mortem examination one patch on the left side of the lower jaw, just below the margin of the alveola, was gangrenous and fully the size of an old-fashioned shilling piece. In the intestinal canal were found about a dozen tape-worms, all of one variety and of various ages and sizes. The anterior segment was peculiar in being bifid, while the blunted extremeties of each head was armed with a single sucker but no hooks. The segments were imperfectly separated from one another, as in all of this special variety of tape-worms. A fine sample has been sent to Mr. Cobbold, of London. The books contain no description of a tape-worm provided with such a peculiar but characteristic head.

CENTRAL PARK, NEW YORK CITY.

